# Non-Destructive Study of Bulk Crystallinity and Elemental Composition of Natural Gold Single Crystal Samples by Energy-Resolved Neutron Imaging

**DOI:** 10.1038/srep40759

**Published:** 2017-01-19

**Authors:** Anton S. Tremsin, John Rakovan, Takenao Shinohara, Winfried Kockelmann, Adrian S. Losko, Sven C. Vogel

**Affiliations:** 1 Space Sciences Laboratory, University of California at Berkeley, 7 Gauss Way, Berkeley, CA 94720, USA; 2 Department of Geology and Environmental Earth Science, Miami University, 250 South Patterson Ave., Oxford, OH 45056, USA; 3 Japan Atomic Energy Agency, 2-4 Shirakata-shirane Tokai-mura, Naka-gun Ibaraki 319-1195, Japan; 4 STFC-Rutherford Appleton Laboratory, ISIS Facility, Didcot, OX11 0QX, UK; 5 Materials Science and Technology Division, Los Alamos National Laboratory, Los Alamos, NM 87545, USA

## Abstract

Energy-resolved neutron imaging enables non-destructive analyses of bulk structure and elemental composition, which can be resolved with high spatial resolution at bright pulsed spallation neutron sources due to recent developments and improvements of neutron counting detectors. This technique, suitable for many applications, is demonstrated here with a specific study of ~5–10 mm thick natural gold samples. Through the analysis of neutron absorption resonances the spatial distribution of palladium (with average elemental concentration of ~0.4 atom% and ~5 atom%) is mapped within the gold samples. At the same time, the analysis of coherent neutron scattering in the thermal and cold energy regimes reveals which samples have a single-crystalline bulk structure through the entire sample volume. A spatially resolved analysis is possible because neutron transmission spectra are measured simultaneously on each detector pixel in the epithermal, thermal and cold energy ranges. With a pixel size of 55 μm and a detector-area of 512 by 512 pixels, a total of 262,144 neutron transmission spectra are measured concurrently. The results of our experiments indicate that high resolution energy-resolved neutron imaging is a very attractive analytical technique in cases where other conventional non-destructive methods are ineffective due to sample opacity.

An impressive number of non-destructive techniques have been developed to date that can provide information about bulk structure and elemental composition of a vast range of materials. In some cases however, conventional methods utilizing X-rays are inadequate because they fail to penetrate deep enough into samples consisting of elements with large atomic numbers. Neutrons, on the other hand, have attenuation cross sections not correlated to, and not increasing with the atomic number, and thus can often be used as probes of the samples where X-ray techniques fail due to their opacity. Neutron diffraction and imaging have provided unique possibilities in the study of microstructures of metal samples^1^,^2^,^3^, the distribution of hydrogen and hydrogen-containing molecules within complex 3D samples, e.g. water distribution in fuel cells^4^, the dynamics of water penetration in geomaterials and other porous media^5^, the distribution of high-Z materials within other high-Z structures, such as in nuclear fuels^6^, mapping organic materials within objects of cultural heritage, such as thick-walled bronze Buddha statues^7^, and many other samples and materials. Most high resolution neutron imaging experiments are conducted at continuous neutron sources without resolving the neutron energies and are mostly limited to thermal and cold spectral energy ranges^8^. In addition to conventional attenuation based imaging, spatially resolved neutron diffraction experiments enable accurate studies of microstructure, texture and strain^9^, but with only limited spatial resolution, requiring scanning through the sample volume with a highly collimated beam and resulting in long experimental acquisition times. Recent developments of high resolution neutron counting detectors combined with bright pulsed neutron sources enable high resolution energy-resolved neutron imaging with mm-scale^10^ and even sub-100 μm spatial resolution^11^,^12^. This new generation of neutron imaging detectors can register neutrons with 10–500 ns timing resolution (varied with the neutron energy), providing the possibility to measure neutron transmission simultaneously over a wide range of energies, from epithermal keV-energy neutrons to cold ~meV neutrons, thus, spanning six orders of magnitude in neutron energy and the corresponding changes in neutron attenuation due to different interaction mechanisms in a single experiment. Energy-resolved neutron imaging is *the only technique utilizing the entire spectrum* available at spallation neutron sources to characterize materials for a broad range of parameters: Interrogation with thermal and cold neutrons can provide information on bulk microstructure, phase, texture, and strain distribution in metal samples *via* Bragg-edge imaging^8^,^10^,^12^. Spatial variations of hydrogen in substances can also be measured *via* “regular” attenuation based imaging (e.g. hydrogen distributions in zirconia samples^13^). At the same time the presence of neutron resonances in the epithermal range of energies can be utilized to map elemental/isotope distributions within samples^6^,^11^,^14^,^15^,^16^. Moreover, simultaneous measurements of transmission together with the diffraction spectra can be performed in one experiment^17^. Recently developed tools for the microstructure analysis of mosaic crystals open up new opportunities for quantitative neutron transmission analysis^18^.

In this study we demonstrate the possibility of mapping bulk microstructure and elemental composition within materials opaque to conventional non-destructive testing techniques. In one experiment we combine thermal and cold neutron diffraction, transmission, and neutron resonance spectroscopy in one relatively simple experimental setup, utilizing a single fast neutron counting detector installed at a pulsed neutron beamline. The internal structures and elemental compositions of two single-crystal and two polycrystalline natural gold samples are mapped in our experiment with ~100 μm spatial resolution. The results of this study demonstrate the unique capabilities of energy-resolved neutron transmission and diffraction imaging for the non-destructive study of nearly cm-thick natural gold samples, which are opaque in conventional X-ray methods.

The principle of energy-resolved imaging utilized in the present study is explained schematically in Fig. 1. A neutron counting detector with 512 × 512 pixels (55 × 55 μm^2^ pixel-size)^19^,^20^, providing both position (*X,Y*) and time-of-flight (*T*) for every detected neutron, was installed at distance *L* = 14.3 m from the neutron spallation target at the NOBORU beamline of the Japan Proton Accelerator Complex (J-PARC). The gold samples were mounted at ~15 mm distance from the detector active area. The non-relativistic energy *E* of each neutron is calculated from its time-of-flight (TOF) *T*, measured by the detector relative to a trigger that is synchronized to the time of spallation


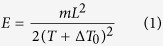


where *m* is the mass of the neutron, and *ΔT*_*0*_ is the time delay of the trigger pulse relative to the time of spallation. Each pixel of the detector operates independently, enabling the detection of tens of thousands of nearly simultaneous events. Multiple neutron pulses were acquired in TOF mode, collecting several thousand time resolved images, with each image corresponding to a specific energy calculated with eq. 1. For the entire active detector area, this results in a total of 512 × 512 = 262,144 transmission spectra measured simultaneously, and spatially resolved with 55 × 55 μm^2^ pixel size. In the cases of insufficient neutron statistics for a single pixel that is required for a quantitative analysis of the sample properties, neighboring pixels can be combined in the post-experimental analysis in order to reduce the statistical fluctuations of measured spectra (e.g. spectra shown in Fig. 1c, where the transmission of sample N3 is integrated over its entire area covered by the detector). To eliminate the effects of spatial heterogeneities of the neutron beam and the non-uniformities of the detector response, all measured transmission spectra were normalized with ‘open beam’ TOF spectra, measured without the sample in the beam path. The accurate value of the distance between the source and the detector *L*, as well as time delay of the source trigger *ΔT*_*0*_ were obtained by a linear fit to the function 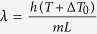, where h is Plank’s constant. The fit to experimental data is shown in Fig. 1d, where the tabulated wavelengths of Au and Pd resonances (from ENDF database^21^) are plotted versus measured time of flight values *T*.

## Samples

**Figure 2:**
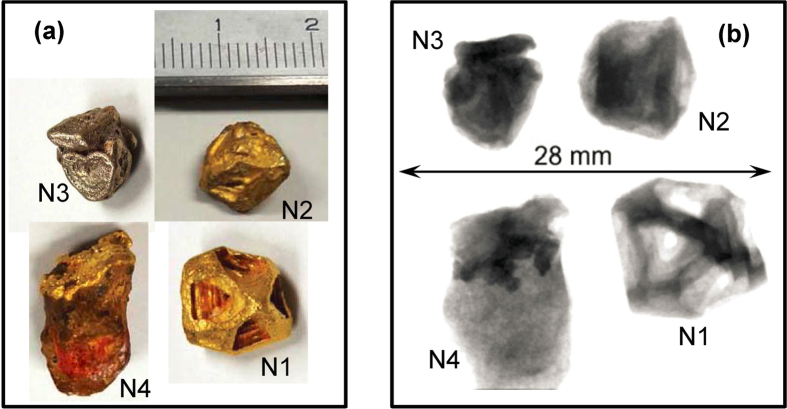
Gold samples measured in the experiment. (**a**) Photographs of analyzed gold samples. (**b**) White beam neutron radiographies of samples. The entire spectrum of the beam is used to form an image.

Four gold samples (N1-N4, Fig. 2), all from elluvial or alluvial placer deposits found west of the Roraima Shield District, Bolívar, Venezuela, were analyzed in this study. Sample N1, from Icabaru, is a morphologically well formed hoppered octahedral crystal, for which the crystallinity was previously studied by X-ray and neutron diffraction methods^3^. The sample is known to have a polycrystalline surface texture but the bulk of the sample is a single crystal (i.e. translationally contiguous). Samples N2 and N3, also from the Icabaru area, visually appear to be morphologic single crystals, but their color indicates areas of Au-Pd alloy (porpezite). Sample N4, from Mina Zapata, Santa Elena de Uairen, is an anhedral mass in a matrix of lateritic sediment. It exhibits morphologic features that suggest that the gold grew in the laterite, i.e. it is authigenic^22^. Its morphology does not indicate a single crystal and it is speculated to be polycrystalline.

## Results

### Mapping of elemental composition through neutron resonance transmission analysis

**Figure 3:**
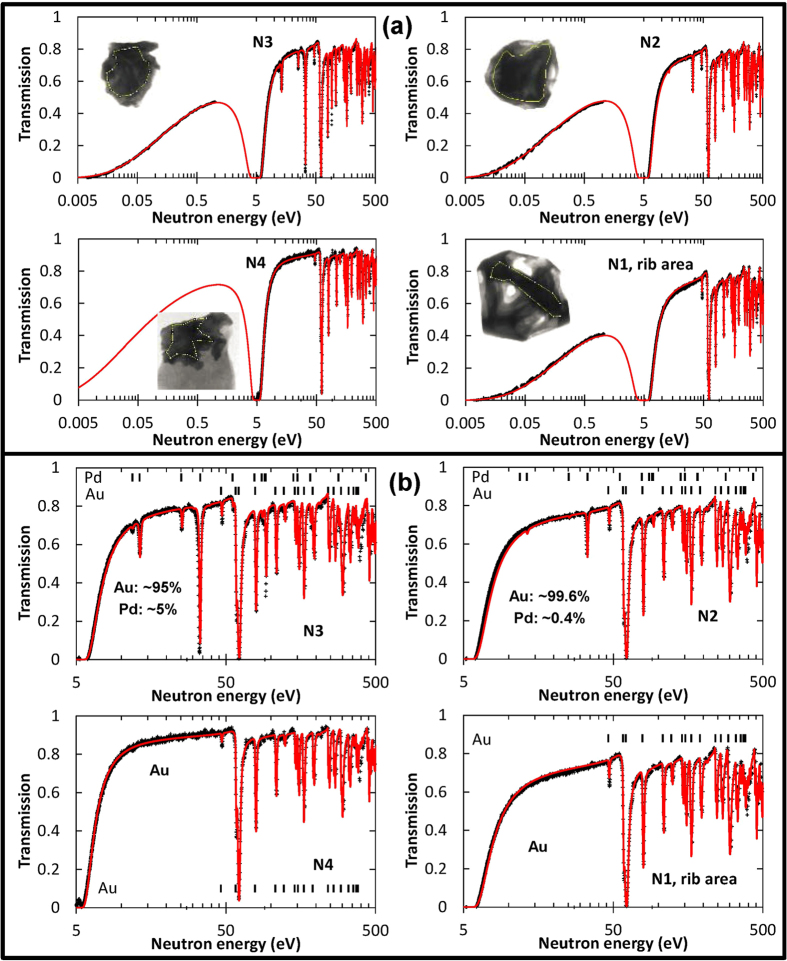
Neutron transmission spectra measured for four gold samples. Symbols are measured data points, solid lines represent theoretical transmission curves calculated from tabulated attenuation cross sections. (**a**) Full spectra showing strong absorption of gold samples in the thermal range of neutron energies (<50 meV). The inserts are full-spectrum transmission images of the gold samples and yellow lines indicate the areas over which transmission spectra were integrated. (**b**) Neutron transmission spectra measured in the epithermal range of energies, indicating the presence of Pd resonances for samples N2 and N3. The legends indicate the atomic percentage concentration of Pd and Au in the samples, obtained by the fit of the calculated spectra to the measured transmission.

The transmission spectra measured for the 4 gold samples are shown in Fig. 3 together with the transmission calculated from tabulated cross sections (ENDF database^21^). The measured spectra are obtained by integration over a relatively large sample area shown by dashed lines on the insets of this figure (Fig. 3). The resonance absorption by certain isotopes contained in the gold samples leads to the formation of sharp transmission dips for energies above ~1 eV, while the gradual decrease of transmission at energies below ~1 eV is mostly due to the off-resonance absorption for thermal neutrons by the gold nuclei. The gap in the measured data for the energy range of ~0.6–5 eV is due to the detector deadtime of 320 μs, required for data readout. Several acquisition periods, separated by readout gaps, were implemented in our experiment for each neutron pulse. The readout sequence resets the entire sensitive area of the detector and reduces detector saturation effects, which can affect the quantification of the element concentrations. The ~0.6–5 eV spectral region was chosen for the readout as a compromise between the measurements of resonance absorption and non-resonant attenuation by thermal and cold neutrons. Quantitative analysis of the elemental composition of the samples is performed by fitting the calculated transmission (assuming natural composition of the isotope ratios for each element) to measured spectra, with the sample thickness and elemental concentration being fitted parameters. Ideally, the fitting procedure would be performed for individual spectra accumulated in each 55 μm pixel, for which the thickness of the sample can be considered nearly constant. However, limited statistics acquired for the spectrum within such a small area in our experiment did not allow for a more robust and accurate fitting analysis. Instead, the spectra averaged over the sample areas indicated in Fig. 3 were used in the present analysis. The thickness of a sample is not constant over these areas; however, it does not vary by a large factor either. Thus, the reconstructed thickness and concentration values should be treated as estimates only, as they are not as precise as they could be, given sufficient counting statistics in each pixel of our dataset. Elements present in the sample were identified by their resonance energies before fitting of the spectra. Elements present in the sample were identified by their resonances energies before fitting of the spectra. It should be noted that only isotopes with relatively strong resonances at energies below ~1 keV can be identified in our current analysis. However, some light or low abundance elements present in the laterite part of sample N4 (e.g. Si, O, Al, Fe) cannot be identified in the data due a lack of absorption resonances in the measured energy range. The best fits were obtained with sample thicknesses of ~5.2 ± 0.2, ~4.6 ± 0.15, ~3.8 ± 0.1 and ~1.9 ± 0.1 mm and Au atomic fractions of ~1, ~0.9957 ± 0.0004, ~0.950 ± 0.005, and ~1 for samples N1, N2, N3 and N4, respectively, with ~0.43 ± 0.04% and ~5 ± 0.5% Pd in samples N2 and N3. The accuracies of reconstructed concentration values strongly depend on the resonance cross sections and the concentrations of the elements present in the sample. Therefore the accuracies achieved by the presented reconstructions cannot be generalized. However, it can be concluded that even small concentrations of elements with relatively high resonance absorption cross sections can be mapped within samples opaque to many conventional non-destructive testing techniques.

**Figure 4:**
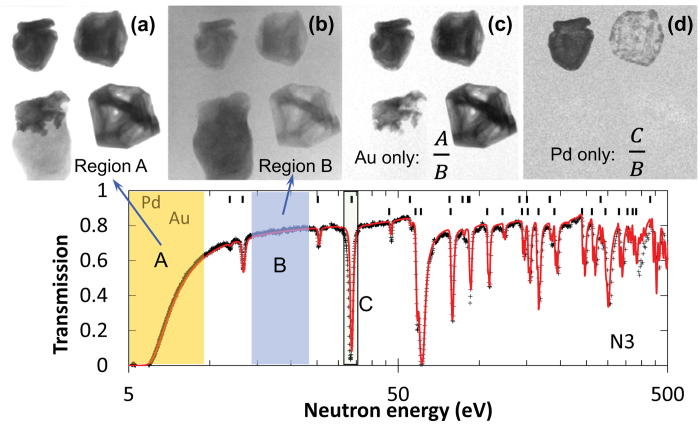
Energy-specific neutron transmission images allowing mapping of the elemental distributions. (**a**) Transmission image obtained at the energies of gold resonance of spectral region A. (**b**) Transmission measured at energies B with no resonances present for these samples. (**c**) Image (**a**) normalized by image (**b**), emphasizing contribution of gold only. (**d**) Image measured at the Pd resonance energy (spectral region C) normalized by an off-resonance region B, emphasizing the distribution of Pd within the samples. No Pd is detected in samples N1 and N4.

To this point it is demonstrated that the bulk elemental composition can be recovered from the measured spectra for elements showing absorption resonances in the accessible neutron energy range. This is enabled by the unique capability of epithermal neutrons to penetrate materials otherwise opaque to conventional non-destructive testing techniques, such as “regular” X-rays and thermal neutron imaging. In addition, the spatial distribution of Au and Pd within the samples can also be reconstructed from the same experimental data, as demonstrated in Fig. 4. A gold-specific transmission image can be obtained by the division of the transmission image of Fig. 4a (taken at the resonance energies of gold) by the image of Fig. 4b, obtained in a region of the spectrum where no resonances are present; shown in Fig. 4c. With this normalization the radiograph contrast resulting from other elements is greatly suppressed and the resulting image maps the spatial distribution of gold nuclei within the samples. Similar normalization, except using Pd resonance energies, produces the image of the Pd distribution within the samples, as shown in Fig. 4d, indicating that the bulk distribution of palladium in samples N2 and N3 is not uniform. Discrete domains of increased Pd concentration are observed in sample N2 and a continuous zone of increased Pd concentration, in the exterior ~2 mm of sample N3. No Pd or other elements with measureable resonances were observed in samples N1 and N4, except for the resonance of the Au nuclei.

### Mapping of bulk crystallinity through Bragg diffraction

Coherent neutron diffraction is used for the study of the structures (atomic arrangement) and microstructures (crystallinity and texture) of crystalline materials. Neutron diffraction occurs for thermal and cold neutron energies for which the corresponding wavelengths of neutrons are comparable to interplanar distances within the crystal structure. In a diffraction experiment, the intensities and shapes of diffraction peaks, formed by neutrons scattered out of the incident beam are used for the reconstruction of texture, strain, and phase compositions of interrogated samples^1^,^2^,^9^. At the same time, coherent neutron scattering leads to the formation of Bragg edges, i.e. sharp increases of sample transmission (in of a polycrystalline sample) and dips (in case of a single crystal) in transmission spectra, which can similarly be used for the study of microstructure properties using neutron transmission data^1^,^10^,^12^,^23^,^24^. In the present experiments we combined the measurements of sample transmission and forward scattered diffraction patterns in order to study the crystallinity of the four natural gold samples, in addition to their elemental composition presented in the previous section.

**Figure 5:**
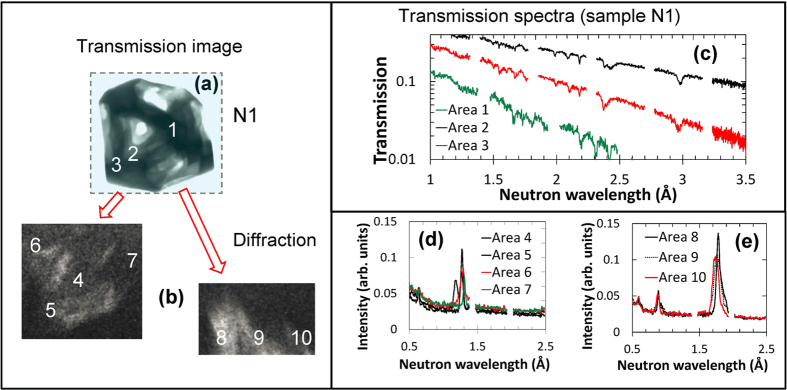
Transmission spectra and diffractograms from sample N1. (**a**) The transmission image and **(b)** two forward diffraction spots measured simultaneously with the transmission image (**a**). The incoming neutron flux was limited to the area indicated by the dashed rectangle. Neutrons forming images (**b**) were diffracted by the sample. The numbers (1–3) indicate the areas for which the transmission spectra are shown in (**c**); numbers (4–10) indicate areas for which the diffraction data are shown in (**d**) and (**e**). The dips in spectra (**c**) and peaks in (**d**) and (**e**) indicate that sample N1 is a single crystal.

**Figure 6:**
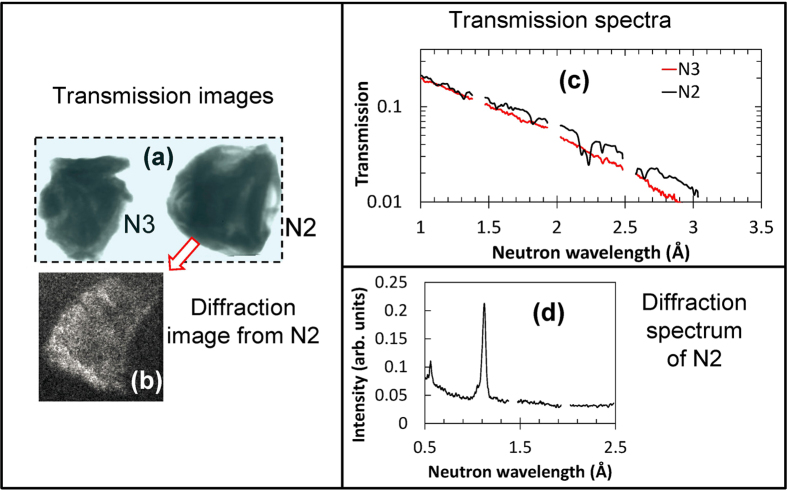
Transmission image, spectra of samples N2 and N3, and diffraction image of sample N2. (**a**) The transmission image and **(b)** forward diffraction spot from sample N2 measured simultaneously. Incoming neutron flux was limited to the dashed rectangle shown in (**a**); all neutrons in image (**b**) were diffracted by sample N2. **(c)** Thermal transmission spectra measured for samples N2 and N3 (integrated over the entire sample area excluding edges). (**d**) Diffraction pattern of the neutron diffraction spot from sample N2. The dips in spectra (**c**) of sample N2 and peaks in (**d**) indicate that sample N2 is a single crystal, while sample N3 exhibits a polycrystalline transmission spectrum (**c**).

A little studied characteristic of naturally-occurring gold samples is their bulk crystallinity, which cannot be examined non-destructively with conventional X-ray or even synchrotron methods for centimeter-scale samples due to their opacity. Thermal and cold neutrons can penetrate such samples (with the limit to a ~centimeter thickness; the half thickness for 100 meV neutrons is about 2 mm) and can reveal the presence of large grains or texture within the sample^3^. Thus, it can distinguish single crystals from polycrystalline samples and can be used to analyze the texture in polycrystalline aggregates. Natural single crystal gold samples of several mm size are rare, and thus non-destructive evaluation of such specimens is of great importance to understand their crystalline structures and, by extension, their formation conditions. Also, because of the malleability of gold, sectioning of samples invariably disturbs their crystallinity. In our experiments we used a single detector for the registration of both the diffracted and transmitted neutrons. One half of the detector was masked by a Cd filter (installed upstream of the gold samples), blocking half of the detector from the direct neutron beam in thermal and cold energy ranges, while the other half was used for the measurement of the transmitted spectra, which are shown in Figs 5 and 6. In this way both energy-resolved transmission and diffraction images were obtained simultaneously for our gold samples. Apparently, only a small fraction of the diffracted signal was registered in our experiment due to a very small active area of the detector. A full 4π coverage is preferred. However, the relatively small detector area used for detection of the diffraction signal was sufficient to assess the crystallinity of the samples.

The shape and distribution of the spots observed in the diffraction images can reveal the presence of multiple large grains if present in the sample, as demonstrated in previous studies^25^. In the case of a perfect single crystal, all areas of the sample scatter neutrons in the same direction for a given neutron energy/wavelength, according to Bragg’s law


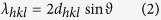


where *λ*_*hkl*_ is the wavelength of neutron, *d*_*hkl*_ is the interplanar spacing for a given set of crystal planes with Miller indices (*hkl*), and 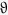 is half the scattering angle. The neutron wavelength can be calculated from its energy *E* according to 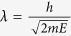. Therefore in the case of a single crystal sample or a sample with a few large grains a narrow-energy (i.e. narrow wavelength range) a diffraction image is formed by the neutrons scattered off a specific set of crystal planes for a corresponding neutron wavelength. The projection of the diffraction spot onto the detector is determined by the grain or single crystal volume within the sample. For a single crystal sample the diffraction spots are formed by the entire sample corresponding to the projection of the entire crystal onto the active detector area. If multiple large grains are present in the sample, each individual grain gives rise to corresponding diffraction spots, revealing its shape and size within the sample volume. Such information potentially enables tomographic imaging of large grains, similar to the 3D X-ray microscope method developed at synchrotrons^26^. The location of a particular grain within the sample can also be found in the transmission images, whereby the corresponding decrease in intensity is due to neutrons scattered away from the direct beam. The angular misalignments between the grains can be reconstructed from the corresponding diffractograms. It has been demonstrated previously how the mosaicity of the sample structure can be reconstructed from such measurements^18^,^23^. The energy resolution of a particular experiment determines the limit of resolvable angular misalignment between the individual grains. For the present experiments with about ~0.5% energy resolution for thermal and cold neutron energies, an angular misalignment of ~0.5 degree or larger could be observed. In the extreme case of a polycrystalline structure with many domains of different structural orientations, discrete diffraction spots would be absent, resulting in Debye-Scherrer rings of diffraction intensity observed in the measured diffraction images. Also, a sharp increase in the transmission spectra (Bragg edges) would be observed in the areas of the sample containing polycrystalline structure at a neutron wavelength equal to twice the lattice spacing.

The transmission image and two diffraction images from sample N1 are shown in Fig. 5a,b, respectively. The transmission spectra (Fig. 5c) and diffractograms (Fig. 5b) indicate that this sample is a single-crystal. Shifts in the diffraction peak wavelength (~0.1 Å, Fig. 5d) observed in different areas of the diffraction image indicate the presence of some finite mosaicity (several degrees, according to Bragg equation 2) within the sample. Similarly, a single crystalline structure, with no measurable mosaicity, was observed for sample N2 (Fig. 6). Sample N3 did not produce any measured diffraction spots. This may simply be due to a small diffraction detection area missing the diffraction spots. However, sample N3 also did not exhibit any dips in the measured thermal transmission spectra, Fig. 6c. This suggests that N3 is polycrystalline. No Bragg edges, which are typical for a polycrystalline sample, were observed for sample N3, resulting from the high attenuation cross section leading to a very low transmission at wavelengths where the Bragg edges are most pronounced (e.g. 4.71 Å for the strongest 111 edge).

## Methods

### Calculation of sample transmission in the resonance regime

The neutron attenuation of a sample with a given elemental composition and thickness can be calculated from the tabulated attenuation cross section values given as a function of neutron energy in the ENDF database^21^. A more detailed analysis can be performed allowing reconstruction of sample temperature from the resonance broadening, as demonstrated in references^27^,^28^. The present study is limited to reconstruction of the elemental composition from the neutron transmission spectra measured at room temperature. As mentioned earlier, only elements with reasonably high attenuation cross sections in the epithermal range can be identified in this analysis, limiting its sensitivity to certain elements (or more specifically, to isotopes of certain elements).

Quantification of the elemental composition was performed in our analysis by fitting the calculated sample transmission to the experimental data. For a given sample thickness and composition (both fitted parameters), the theoretical sample transmission was obtained from the tabulated attenuation cross sections for the natural isotope abundance. In the case of an ideal neutron beam (with an infinitely short neutron pulse, perfectly parallel trajectories and no background contributions), the transmission for a given neutron energy can be calculated from the equation


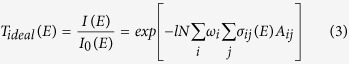


where *l* is the thickness of the sample, *N* is the number of atoms per unit volume, *ω*_*i*_ is the atomic fraction of the *i-th* element, *σ*_*ij*_*(E)* is the neutron attenuation cross section for the *j-th* isotope of element *i*, and *A*_*ij*_ is the abundance of isotope *j* for the element *i*. The number of atoms per unit volume can be obtained from the equation


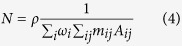


where *ρ* is the sample density and *m*_*ij*_ is the atomic mass of isotope *j* of element *i*. The finite time-width of the neutron pulse changes the measured sample transmission and broadens the resonances, especially at higher energies where the width of the neutron pulse becomes comparable or larger than the width of neutron resonances


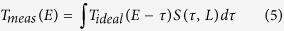


where *S(τ, L)* is the function describing the shape of neutron pulse as a function of neutron energy for a given distance *L* from the source. The shape of the neutron pulse at a given spallation facility is determined by the proton pulse and the moderator used to modify neutron energies. In our analysis the function *S(τ, L)* was approximated by a Cole-Windsor function with parameters corresponding to the NOBORU beamline described in reference^29^.

## Conclusion

The high resolution energy-resolved neutron imaging employed in this study demonstrates a unique possibility to investigate bulk properties of samples that are opaque to other non-destructive testing techniques. It is the combination of fast neutron counting detectors and collimated bright neutron beams available at spallation neutron sources that now allow measurement of spatially-resolved neutron transmission in a wide range of energies, spanning from epithermal neutrons (where neutron resonance absorption provides information on the elemental composition) to thermal and cold neutrons (where Bragg scattering is used for the studies of crystallographic properties of a sample). It is important to note that different bulk material properties can be reconstructed from one measurement and with a ~100 μm spatial resolution. At present, such experiments can be conducted at very few facilities only. The unique capability of neutrons to penetrate objects opaque to other more conventional interrogation methods make these studies very attractive for valuable samples, which can only be studied non-destructively (e.g. rare geological samples, celestial objects or articles of cultural heritage, and others).

## Additional Information

**How to cite this article**: Tremsin, A. S. *et al*. Non-Destructive Study of Bulk Crystallinity and Elemental Composition of Natural Gold Single Crystal Samples by Energy-Resolved Neutron Imaging. *Sci. Rep.* **7**, 40759; doi: 10.1038/srep40759 (2017).

**Publisher's note:** Springer Nature remains neutral with regard to jurisdictional claims in published maps and institutional affiliations.

## Figures and Tables

**Figure 1:**
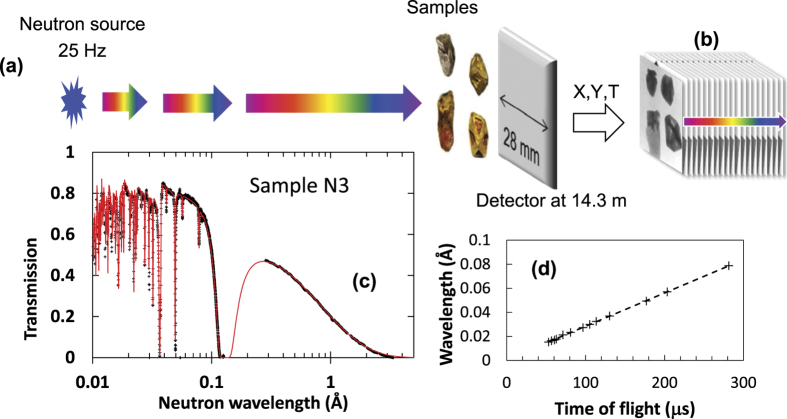
Schematic diagram of experimental setup at a pulsed neutron source. (**a**) Neutron pulses travel from the source towards the sample and detector, which measures position X,Y and time T (determined relative to the time of spallation) for each registered neutron. (**b**) The measurement result is a set of neutron transmission images, each corresponding to a specific neutron energy. (**c**) Neutron transmission spectra measured for one of the samples (N3), extracted from the set of neutron transmission images; the solid line is the transmission calculated from the tabulated cross sections of Au and Pd. (**d**) Calibration of the flight path *L* and the source trigger delay *ΔT*_*0*_.
